# SH3BGRL Suppresses Liver Tumor Progression through Enhanced ATG5-Dependent Autophagy

**DOI:** 10.1155/2023/1105042

**Published:** 2023-04-24

**Authors:** Abdulmomen Ali Mohammed Saleh, Farhan Haider, Haimei Lv, Bin Liu, Jing Xiao, Mingming Zhang, Yuzhen Zheng, Shulan Yang, Haihe Wang

**Affiliations:** ^1^Center for Translational Medicine, The First Affiliated Hospital, Sun Yat-sen University, Guangzhou 510080, China; ^2^Department of Biochemistry, Zhongshan School of Medicine, Sun Yat-sen University, Guangzhou 510080, China; ^3^Zhuhai Interventional Medical Center, Zhuhai Precision Medical Center, Department of Clinical Laboratory, Zhuhai People's Hospital (Zhuhai Hospital Affiliated with Jinan University), Zhuhai 519000, China; ^4^Department of Thoracic Surgery, The Sixth Affiliated Hospital, Sun Yat-sen University, Guangzhou 510655, China

## Abstract

SH3BGRL, an adaptor protein, is upregulated in breast cancers and indicates its tumorigenic role. But the function of SH3BGRL in other types of cancers is largely unknown. Here, we modulate SH3BGRL expression level in two liver cancer cells and conduct both in vitro and in vivo analyses of SH3BGRL in cell proliferation and tumorigenesis. Results demonstrate that SH3BGRL notably inhibits cell proliferation and arrests the cell cycle in both LO2 and HepG2 cells. Molecularly, SH3BGRL upregulates the expression of ATG5 from proteasome degradation as well as the inhibitions of Src activation and its downstream ERK and AKT signaling pathways, which eventually enhance autophagic cell death. The xenograft mouse model reveals that SH3BGRL overexpression can efficiently suppress tumorigenesis in vivo, while the additional silencing ATG5 in SH3BGRL-overexpressing cells attenuates the inhibitory effect of SH3BGRL on both hepatic tumor cell proliferation and tumorigenicity in vivo. The relevance of SH3BGRL downregulation in liver cancers and their progression is validated based on the large-scale tumor data. Taken together, our results clarify the suppressive role of SH3BGRL in tumorigenesis of liver cancer, which would be of help to the diagnosis of liver cancer, while either promoting the autophagy of liver cancer cells or inhibiting the downstream signaling induced from SH3BGRL downregulation would be a promising therapy.

## 1. Introduction

Liver cancer is one of the most lethal causes of cancer death worldwide. More than 840,000 new liver cancer cases and 780,000 cancer deaths from liver cancer are reported each year, and the trends are still increasing [1, 2]. Hepatocellular carcinoma (HCC) is the most frequent primary liver cancer, accounting for 80%–90% of all cases [3, 4], but the underlying causes of tumorigenesis and progression are still obscure.

SH3BGRL, an adaptor protein, is one member of the SH3BGR family, in which another three members, SH3BGR, SH3BGRL2, and SH3BGRL3 are included [5]. SH3BGRL is ubiquitously expressed in varied human tissues and organs, including the bone marrow, heart, lung, liver, and kidney [6]. We also thoroughly characterized the mRNA expression pattern of SH3BGR family members during zebrafish embryo development and found that *sh3bgrl* mRNA is dynamically expressed during development, and confined to the intestine at the adult stage [7]. SH3BGRL encodes a small protein with a conserved proline-rich PLPPQIF region and two motifs, Homer EVH1-binding and SH3-binding motifs [8]. As a scaffold protein, SH3BGRL could participate in the protein-protein interaction for the integration or/and crosstalk of signal transduction, membrane trafficking, cytoskeletal rearrangements, and other key cellular processes [9].

We previously uncovered that mouse SH3BGRL (mSH3BGRL) drives colorectal cancer metastasis through c-Src activation, but the human SH3BGRL function as a tumor suppressor in triple-negative breast cancers [10]. Moreover, we also verified the suppressive role of human SH3BGRL in leukemogenesis [11]. However, a clinical study demonstrated that SH3BGRL is highly expressed in breast tumors and squamous oral carcinomas, indicating its possible tumor-facilitating function [9, 12, 13]. As SH3BGRL was predicted to bind with HER2 [14], we then figured out the exact function of SH3BGRL in HER2-positive breast cancers and revealed that SH3BGRL can efficiently bind with HER2, which subsequently activates the downstream signals to enhance the HER2-targeted drug resistance [15]. All the above study indicates that SH3BGRL may function through the cell type or context-dependent manner in tumor progression. However, there is no more information about its role in other types of cancer.

To expand and explore the exact function of SH3BGRL in more types of cancers, including liver cancer progression, here we tentatively characterize the physiological role of SH3BGRL in liver cancer cells and unveil one of the unprecedented roles of SH3BGRL in liver cancer cells to enhance apoptosis through interaction to stabilize ATG5 for autophagy-mediated cell death.

## 2. Materials and Methods

### 2.1. Cell Culture

Human normal liver cell line LO2 and the liver cancer cell line HepG2 (HB-8065) were purchased from The Cell Bank of Type Culture Collection of the Chinese Academy of Sciences (Shanghai, China) and American Type Culture Collection (Manassas), respectively. Cells were maintained in DMEM medium (Gibco) supplemented with 10% heat-inactivated fetal bovine serum (FBS) (PAA Laboratories GmbH, Austria) and 1% antibiotics (Sigma) at 37°C with 5% CO_2_.

### 2.2. Cell Transfection and Stable Cell Pools

p-EGFP-C1-SH3BGRL vector-encoding overexpression of SH3BGRL and p-EGFP-C1 control was transfected into HepG2 and LO2 cells using Lipofectamine 2000 (Invitrogen) as previously described [14]. Transfected cells were selected 18 h later with G418 for 2-3 weeks. Fluorescent cells were subsequently sorted from nonfluorescent cells using Flow Cytometry Sorter (MoFlo Astrios EQs, Beckman Culter Life Sciences) to obtain stable cell pools. For knockdown of SH3BGRL in HepG2 and LO2 cells, two shRNA constructs against SH3BGRL (OriGene; Cat#TG309466) were transfected to establish HepG2 and LO2 SH3BGRL stable knockdown cell pools. Similarly, two specific shRNA constructs containing the core target sequences (5′-GCATCTGAGCTACCCAGATAA-3′ and 5′-CCTTGGAACATCACAGTACAT-3′, respectively) against ATG5 or scrambled RNAs were synthesized and inserted into the BamHI and HindIII linearized GFP-V-RS shRNA-29 expression vector (OriGene), which were subsequently transfected into cells to establish stable cell line pools with ATG5 knockdown and control cells.

### 2.3. Cell Proliferation, Cytotoxicity, and Cell Cycle

The growth rate and cytotoxicity of cells were evaluated by using the CCK-8 cell proliferation kit (Dojindo Laboratories, Japan), according to the manufacturers' instructions. Cell-cycle analysis was carried out by flow cytometry with a flow cytometer (Beckman) after propidium iodide staining.

### 2.4. Antibodies

Primary antibodies against GFP (#2956), p-Erk1/2(#4965), MEK (#9126), p-MEK (#9121), AKT (#9272), p-AKT 473 (#9271), p-Src 416 (#2010), p-Src 527 (#2105), ATG5 (#8540), LC3B (3868), P62 (# 2631), *β*-Actin (#4967), and Src (#2109) were from Cell Signaling Technology. Antibodies against ERK1/2 (Santa Cruz, sc-292838) and anti-GAPDH (Millipore, MAB374) were also used as a loading control. Anti SH3BGRL (sc-377108) were purchased from Santa Cruz Biotechnology.

### 2.5. Western Blotting

Cells with 70–85% of confluency were washed with ice-cold PBS and lysed in cold lysis buffer (10 mM Tris-HCL, pH 7.4, 150 mM NaCl, 1% Triton X-100, 0.5% NP-40, 1 mM EDTA, 0.2 mM Sodium Orthovanadate, 0.2 mM PMSF, and protease inhibitor cocktail) on ice for 30 min. Total lysates were collected with rubber scrapers, transferred to a 1.5 ml tube on ice, and then centrifuged at 12,000*g* for 15 min at 4°C. Tumor lysates were extracted similarly except minced with tissue homogenizer to grind the tissues in cold lysis buffer on ice. Total protein concentration was determined by Bio-Rad protein assay (Bio-Rad). Thirty to forty micrograms of protein from each sample were separated. For western blotting, total lysates or IP elutes were run on a 10 to 12% SDS-PAGE gel, and transferred onto a nitrocellulose membrane (Amersham). The blots were blocked in 5% skim milk in PBS with 0.01% Tween 20 for 2 h. After incubation with primary antibodies for 2–4 h at room temperature or overnight at 4°C in a refrigerator on an orbital shaker, blots were washed with TBS-T and incubated with secondary antibody HRP-labeled antirabbit (Cell Signaling Technology) or antimouse antibody (GE Healthcare Life Sciences) for 1 h at room temperature. An enhanced chemiluminescence kit (ECL, Pierce) was used for the final band detection. Western blots were scanned with densitometry and analyzed by ImageJ software to indicate the relative protein expression level from 3 independent experiments, compared to the loading controls.

### 2.6. Immunohistochemistry

Mouse xenograft tumors were dissected, formalin-fixed and paraffin-embedded, followed by sectioning and detection with antibodies against SH3BGRL (Clone 246). The EnVision Systems K 1395 (Dako) was utilized to perform IHC analysis as previously described [16].

### 2.7. Immunoprecipitation

Cells grown in culture dishes were lysed with an appropriate volume of lysis buffer (25 mM HEPES (pH 7.4), 150 mM NaCl, 1% NP-40, 1 mM EDTA, and 1 mM phenylmethylsulfonyl fluoride (PMSF)) on ice for 10 min, followed by clarification with microcentrifugation. The supernatants were incubated with 25 *μ*L of antibody or IgG-cross-linked protein G magnetic beads overnight at 4°C. The magnetic beads were then washed four times with wash buffer (25 mM HEPES (pH 7.4), 150 mM NaCl, 0.5% NP-40, and 1 mM EDTA, 1 mM PMSF). After removing all the liquid, the pelleted beads were resuspended in 1 M glycine (pH 3.0) and denatured for electrophoresis separation on SDS-PAGE and immunoblot analysis.

### 2.8. Immunofluorescence Staining

Cells were fixed with 4% paraformaldehyde and then permeabilized with 0.5% Triton. Samples were blocked with 1% BSA for 30 min at room temperature and stained with mouse anti-SH3BGRL monoclonal antibody overnight at 4°C, followed by incubation for 1 h at room temperature with fluorescein (FITC)-conjugated goat antimouse secondary antibody (Santa Cruz Biotechnology) in the dark. Finally, the samples were mounted with an antifade reagent with DAPI (Invitrogen, Carlsbad, CA). Imaging was conducted with a fluorescence microscope (Nikon, Japan).

### 2.9. Xenograft Tumor Models in Immunodeficient Mice

Four-week-old nude mice were purchased from the Experimental Animal Center of Sun Yat-sen University and utilized for the *in vivo* experiments. 1 × 10^6^ SH3BGRL-overexpressing cells, its knockdown cells or the cells with additional ATG5 knockdown cells with their corresponding parental control cells were subcutaneously injected into the flanks of each nude mouse, respectively. After 28 days, mice were sacrificed and photographed and tumor weight was scored and analyzed. All experiments using nude mice were strictly performed following the guidelines of the Institutional Animal Care and Use Committee (IACUC) at Sun Yat-sen University.

### 2.10. Statistical Analysis

For the xenograft tumor formation assay, the paired Student's *t*-test was used to test the significant difference in tumor weight. Statistical analysis was done using the SPSS 15.0 software package (IBM), and *p* values <0.05 were considered statistically significant.

## 3. Results

### 3.1. SH3BGRL Represses Liver Cancer Cell Cycle Progression

**Figure 1:**
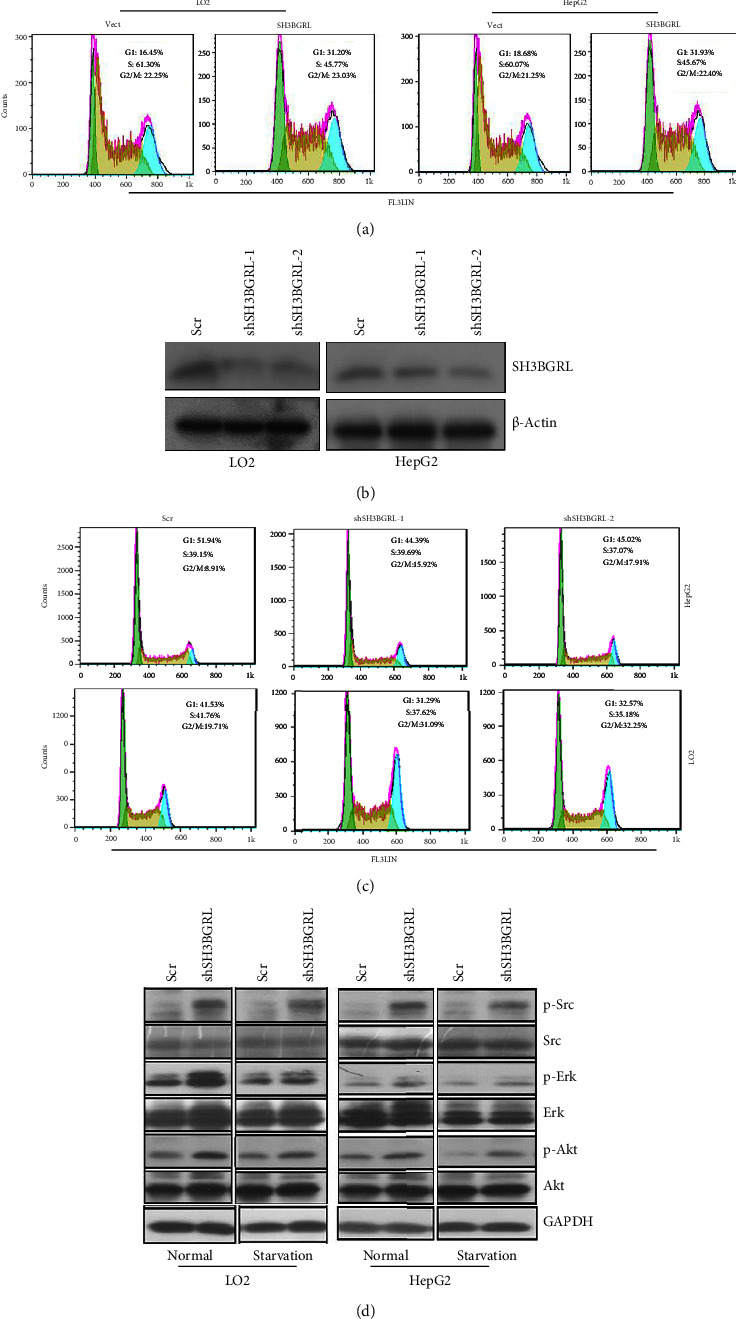
SH3BGRL arrests liver cell cycle progression: (a) flow cytometry analysis of cell cycle progression of liver cancer cells LO2 and HepG2 with SH3BGRL overexpression, compared with the control cells (Vect). Cells were grown at 50%–70% of confluency and starved for 12 h, then cultured with the full medium in less than 12 h. (b) Immunoblots of endogenous SH3BGRL by knockdown with two specific shSH3BGRL-1 and -2. (c) Flow cytometry of the indicated pool cells with SH3BGRL knockdown (shSH3BGRL-1,2) as well as their scramble infection control cells (Scr) as in (a). (d) Immunoblots of the indicated proteins in cells with SH3BGRL knockdown. Cells were cultured in either nutrient-sufficient or deficient conditions for 14 hours, followed by immunoblotting analysis.

To demonstrate the exact function of SH3BGRL in liver cancer cell proliferation, we first overexpressed GFP-conjugated SH3BGRL (SH3BGRL) in both LO2 and HepG2 cell lines along with the empty vector (vector), respectively. The flow cytometry cell cycle analysis revealed that overexpression of SH3BGRL arrested the cell cycle at G1 phase, and the cell population increased from 16.45 to 31.20% and 18.68 to 31.93% in both LO2 and HepG2 cells, respectively (Figure 1(a)). In contrast, we knocked down the endogenous SH3BGRL expression with two specific shRNAs in both LO2 and HepG2 cells (Figure 1(b)), and the immunoblots indicated that SH3BGRL clearly promoted the cell cycle progression, showing as more cells entered into the G2/M phase (Figure 1(c)). Given human wild-type SH3BGRL was shown as a tumor repressor in a breast cancer cell line by inactivation of FAK-Src pathway [10], we examined the Src activation situation and the downstream AKT and MAPK signaling pathways and observed that knockdown of SH3BGRL evidently activated Src as well as the downstream PI3K-AKT and MAPK signaling pathways, under both normal cultural condition and starvation (Figure 1(d)). Overall, these results indicated that SH3BGRL might be a tumor suppressor in liver cancer progression.

**Figure 2:**
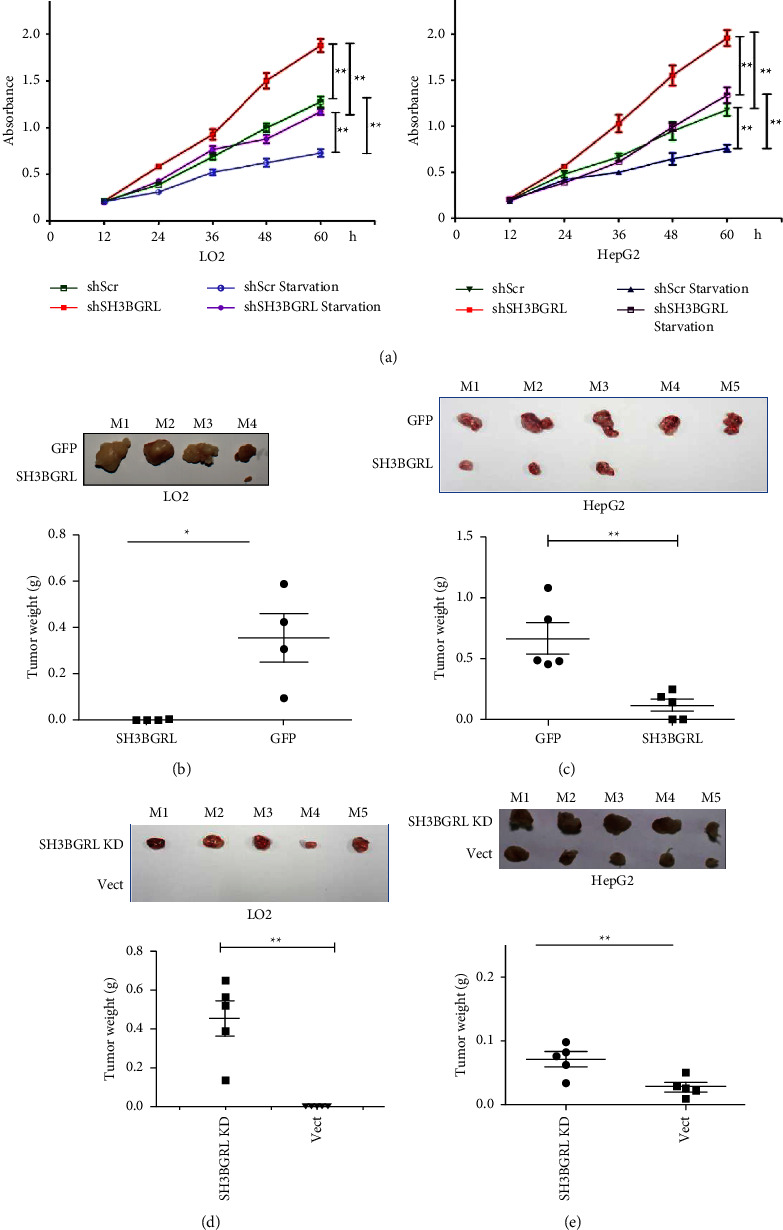
SH3BGRL represses tumor formation of liver cells: (a) CCK-8 assay of the indicated cells with SH3BGRL knockdown under both nutrient-sufficient and deficient conditions. Data present as mean ± SD; *n* = 3; ^*∗∗*^*p* < 0.01. (b, c) Xenograft tumor formation in nude mice by subcutaneous inoculation of 1 × 10^6^ LO2 cells (b) or HepG2 cells (c) with SH3BGRL overexpression, along with their parental control cells (GFP). Cells were injected into the flanks of nude mice. After 28 days, mice were sacrificed and the formed tumor weight was scored. Mean ± SD; *n* = 5; ^*∗∗*^*p* < 0.01; paired Student's *t-*test. (d, e) Xenograft tumor formation in nude mice by inoculation of either LO2 cells (d) or HepG2 cells (e) with SH3BGRL knockdown and the scrambled control (Scr) as mentioned above. Mean ± SD; *n* = 5; ^*∗∗*^*p* < 0.01; unpaired Student's *t-*test.

To test this hypothesis, we determine the physiological function of SH3BGRL in liver cancer cells by CCK-8 cell proliferation assay. Our results manifested that knockdown of SH3BGRL in both LO2 and HepG2 cells could efficiently enhance cell proliferation under both nutrient-sufficient and deficient conditions (Figure 2(a)). To directly confirm the suppressive role of SH3BGRL in liver cancer progression, we approached the xenograft tumor model in nude mice. As expected, overexpression of SH3BGRL dramatically delayed the tumor formation induced by both LO2 and HepG2 cells through subcutaneous inoculation (Figures 2(b) and 2(c)). Conversely, knockdown of SH3BGRL markedly enhanced the tumor formation of both tumor cells (Figures 2(d) and 2(e)). Taken together, our results indicated that SH3BGRL functions as a tumor suppressor in liver cancer progression.

### 3.2. SH3BGRL Promotes Liver Cancer Cell Autophagy

**Figure 3:**
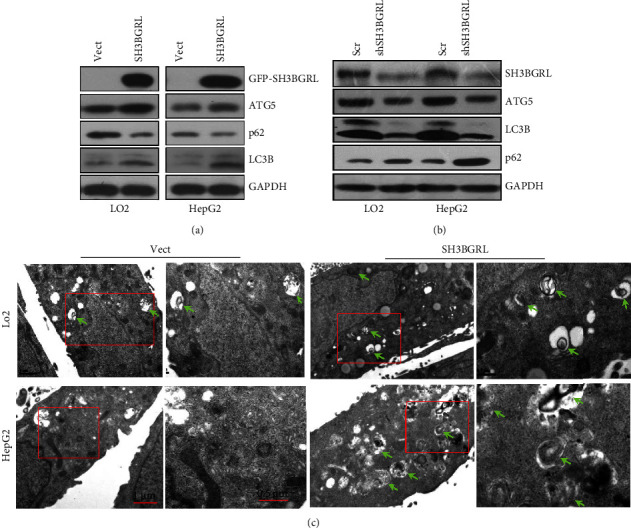
SH3BGRL triggers basal autophagy of liver cells: (a, b) immunoblots of SH3BGRL and the autophagy-related proteins in cells with SH3BGRL overexpression (a) or endogenous SH3BGRL knockdown. *β*-actin was used as a loading control. GFP-vector (vector) and scrambled control (Scr) are shown. (c) Transmission electron microscopy of the formation of autophagosomes in LO2 and HepG2 cells with SH3BGRL overexpression. Enlarged images of the box are shown in the right panels. Representative images of double-membrane autophagosomes are pointed with arrows.

Given that SH3BGRL could inhibit cell proliferation of liver cancer cells in starvation and the documentation of the interaction of SH3BGRL with ATG5 [17], we tentatively checked whether SH3BGRL involves in the autophagy of liver cancer cells, as ATG5 plays a crucial role in autophagy. Therefore, we examined the basal level of autophagy in both LO2 and HepG2 cells with SH3BGRL overexpression. Immunoblots showed that even under the normal culture condition, SH3BGRL could enhance the basal level of autophagy, which is reflected by the elevated conversion level of lipidated LC3B II from LC3B I, as LC3B II can easily associate with the autophagosomal membrane (Figure 3(a)). As p62 is one of the typical cargo receptor proteins that are generally degraded through the autophagy-mediated autolysosomal degradation via binding to LC3B II [18], the lack of p62 degradation in cells with evident LC3B I to LC3B II transition is taken as the incompletion or inhibition of autophagy. Our results here clearly demonstrated that SH3BGRL overexpression eventually rendered p62/SQSTM1 downregulation or degradation. We also observed that an upstream key player for autophagy occurrence, ATG5 was particularly upregulated by SH3BGRL overexpression in cells (Figure 3(a)). Likewise, knockdown of SH3BGRL could abrogate the upregulation of ATG5 and the conversion of LC3B I to LC3B II, while preventing p62 degradation in both cell lines (Figure 3(b)).

To visually and directly verify the autophagy-promoting role of SH3BGRL, we performed the transmission electron microscopy (TEM) analysis of the effect of SH3BGRL on autophagy occurrence in HepG2 and LO2 cells. TEM observation revealed that SH3BGRL overexpression dramatically induced the double-membrane vesicles that resemble the autophagic lysosomes, compared to the rare ones in the control cells (Figure 3(c)), validating our hypothesis. Taken together, these results suggest that SH3BGRL plays a promoting role in the autophagy occurrence in liver tumor cells.

### 3.3. SH3BGRL Binds with the ATG5-ATG12 Complex for Autophagy Progression

**Figure 4:**
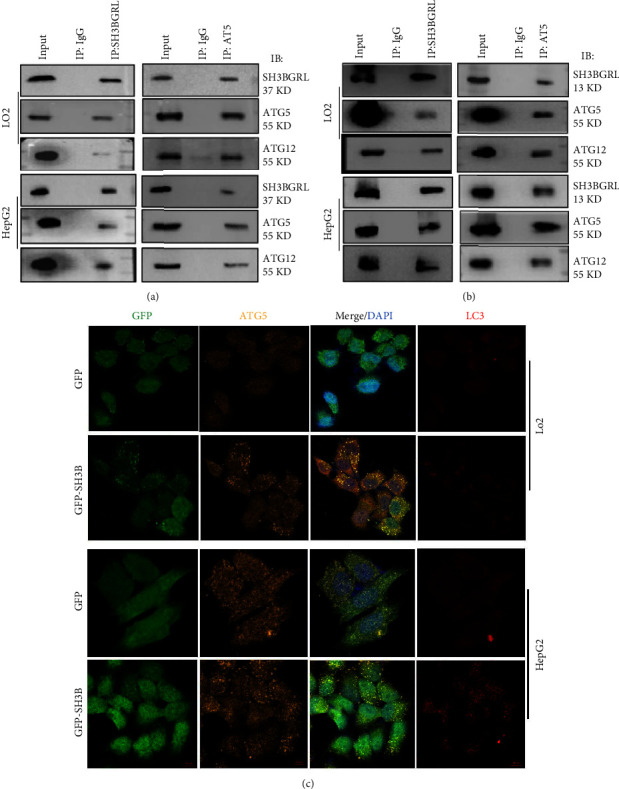
SH3BGRL colocalizes with the ATG5-ATG12 complex: (a, b) mutual immunoprecipitation of SH3BGRL with ATG5 in both LO2 and HepG2 cells with stable overexpression of SH3BGRL (a) or the parental normal cells with SH3BGRL and ATG5 antibodies, respectively. ATG5 and ATG12 were detected at their covalent complex position to indicate autophagy formation. (c) Immunofluorescence of the colocalization SH3BGRL with ATG5 in both LO2 and HepG2 cells with stable overexpression of SH3BGRL or the parental normal cells (GFP) with SH3BGRL and ATG5 antibodies, respectively. LC3 was stained to show its puncta to indicate the autophagy formation.

Given that SH3BGRL upregulates ATG5, we expected if SH3BGRL binds with ATG5 to stabilize ATG5 to trigger autophagy occurrence of liver cells, as SH3BGRL is known as an adaptor protein without any enzymatic activity. To validate this hypothesis, we carried out a mutual coimmunoprecipitation with either SH3BGRL or ATG5 antibodies in SH3BGRL-overexpressing cells. Immunoblots showed that SH3BGRL indeed could be coimmunoprecipitated with ATG5 and ATG12 in both LO2 and HepG2 cells, in which ATG5 and ATG12 were detected at the equal molecular weight of 55KD that is the covalent conjugate of ATG5-ATG12 complex (Figure 4(a)), indicating SH3BGRL is directly involved in the ATG5-related autophagy progression. To clarify whether the endogenous SH3BGRL interacts with ATG5 for autophagy initiation, we also conducted a similar mutual coimmunoprecipitation analysis and found that the endogenous SH3BGRL similarly interacted with the ATG5-ATG12 complex (Figure 4(b)), verifying the involvement of SH3BGRL in promoting autophagy of liver cancer cells, even in the nutrient-sufficient situation. Moreover, we performed the confocal microscopy colocalization visualization and observed that SH3BGRL really manifested the partial colocalization with ATG5 in the cytosol, accompanying the evident LC3 puncta aggregation (Figure 4(c)). Thus, our results further demonstrated that SH3BGRL enhances autophagy in hepatic cancer cells.

### 3.4. SH3BGRL Stabilizes ATG5 by Inhibiting Its Ubiquitination-Mediated Degradation

**Figure 5:**
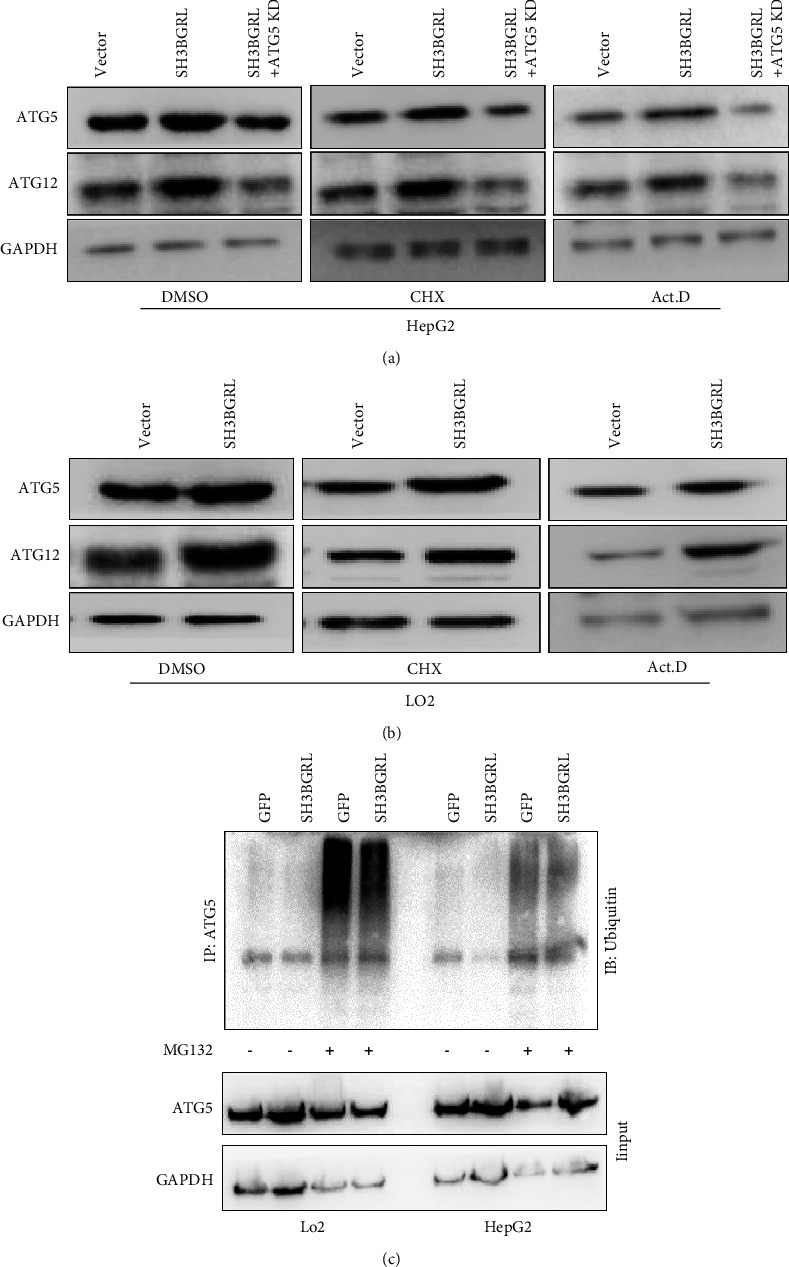
SH3BGRL stabilizes ATG5 from ubiquitination-mediated degradation: (a, b) immunoblots of ATG5 and ATG12 in LO2 cells with SH3BGRL overexpression or knockdown (a) or in HepG2 cells overexpressing SH3BGRL (b) with CHX or Act.D treatments. (c) Ubiquitination of ATG5 analysis by ATG5 antibody immunoprecipitation and immunoblotting in SH3BGRL-overexpressing HepG2 cells. MG132 was used to block ATG5 proteasome degradation.

As known the adaptor feature of SH3BGRL in protein-protein interaction, we speculated that SH3BGRL may bind with either ATG5 or ATG12 for the availability of the ATG5-ATG12 complex in the process of autophagy. Thus, we respectively determined the individual protein stability of ATG5 and ATG12. Our results showed that SH3BGRL overexpression efficiently increased both ATG5 and ATG12 protein levels even by blockade of either the new RNA transcription by Act.D or the novel protein synthesis with CHX, while knockdown of SH3BGRL reversed their protein levels in HepG2 cells (Figure 5(a)). Similar results were also obtained in LO2 cells with SH3BGRL knockdown (Figure 5(b)). All these abovementioned results suggest that the interaction of SH3BGRL with the ATG5-ATG12 complex stabilizes this complex from degradation.

To further dissect SH3BGRL directly stabilize which protein in the complex, we checked the ubiquitination status of both ATG5 and ATG12 through immunoprecipitation of ATG5 or ATG12 with their specific antibodies, followed by immunodetection with ubiquitin antibody. Our results demonstrated that SH3BGRL inhibited the ubiquitination of ATG5, but not ATG12 under inhibition of proteasome degradation by MG132 treatment in both cells (Figure 5(c)). Therefore, we concluded that SH3BGRL may interact with ATG5 to prevent its ubiquitination-related degradation to enhance the consequent autophagy of liver cancer cells.

### 3.5. SH3BGRL Inhibits Liver Tumor Progression through ATG5

**Figure 6:**
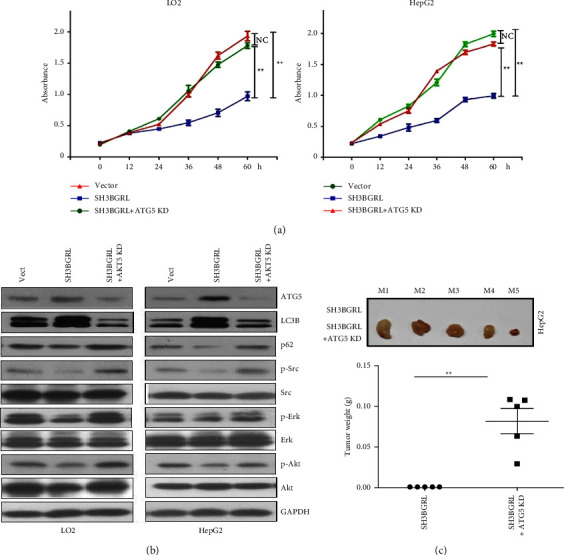
ATG5 mediates SH3BGRL-induced tumor suppression: (a) CCK-8 assay of the indicated cells with additional ATG5 knockdown in SH3BGRL overexpression cells as indicated. Data present as mean ± SD; *n* = 3; ns: no significance; ^*∗∗*^*p* < 0.01. (b) Immunoblots of the indicated proteins in SH3BGRL-overexpressing cells with additional ATG5 knockdown. The parental control cells (Vect) were used. (c) Xenograft tumor formation in nude mice by subcutaneous inoculation of HepG2 cells with additional ATG5 knockdown in SH3BGRL-overexpressing cells. Cells were injected into the flanks of nude mice. After 28 days, mice were sacrificed and the formed tumor weight was scored. Mean ± SD; *n* = 5; ^*∗∗*^*p* < 0.01; paired Student's *t-*test.

To confirm the role of this SH3BGRL-ATG5 axis in liver tumor progression, we additionally silenced ATG5 in SH3BGRL-overexpressing LO2 and HepG2 cells and observed that the additional ATG5 knockdown almost neutralized the inhibitory effect of SH3BGRL overexpression on cell proliferation in both cell lines (Figure 6(a)). Furthermore, the supplemental ATG5 knockdown effectively abolished the SH3BGRL-induced autophagy and the elevated Src and its downstream ERK and AKT activation (Figure 6(b)). To validate the bridging function of ATG5 to SH3BGRL-exerted tumor suppression, we approached the xenograft tumor formation and verified that the extra depletion of ATG5 remarkably counteracted the repressive role of SH3BGRL in tumorigenesis of HepG2 cells, compared to the SH3BGRL-overexpressing cells that induced no tumor formation at all (Figure 6(c)), confirming the mediator function of ATG5 in SH3BGRL-rendered tumor suppression.

### 3.6. SH3BGRL Is Downregulated in Liver Cancer and Is Positively Related to the Basal Level of Autophagy

**Figure 7:**
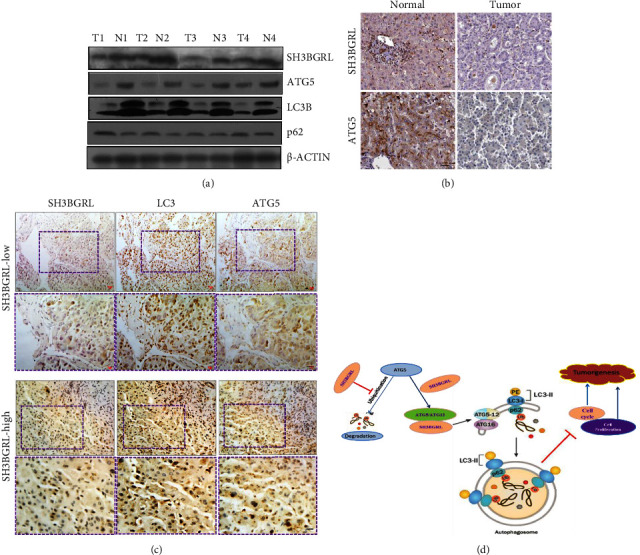
Relevance of SH3BGRL-ATG5 autophagy in liver cancer: (a) immunoblots of SH3BGRL and the indicated autophagy-related proteins in fresh liver tumor samples (T) and their adjacent normal tissues (N). *β*-actin was used as a loading control. (b) Immunohistochemistry of SH3BGRL and ATG5 from The Protein Atlas database (details are indicated in Supplementary Figure S1C). The bar is equal to 50 *μ*m. (c) Immunohistochemistry of SH3BGRL, LC3, and ATG5 in the xenograft mouse tumors in Figure 6(c). Typical SH3BGRL-low and -high tumors are serially sliced. The bar is equal to 25 *μ*m. (d) Schematic model of SH3BGRL-ATG5-autophagy signaling in liver cancer repression.

To validate the physiological role of SH3BGRL-ATG5 in autophagy to inhibit liver tumor progression, we first detected the expression of endogenous SH3BGRL among LO2, HepG2, and SMCC 7721 cells. The semiquantitative PCR showed that the expression of SH3BGRL was downregulated in higher tumorigenic liver cancer cells (tumorigenic potential: LO2 < HepG2 < SMMC7721) (Figure S1A). To verify the authentic relevance of SH3BGRL and autophagy in liver cancer, we collected 4 pairs of fresh liver tumor tissues and detected both mRNA and protein levels of SH3BGRL. Semiquantitative RT-PCR results showed that SH3BGRL was downregulated in all tumor tissues, compared to their corresponding adjacent normal counterparts (Figure S1B). Immunoblots also manifested the consistent SH3BGRL protein downregulation to its mRNA in tumor samples, while the basal level of autophagy in these liver cancers was also positively related to the expression of SH3BGRL (Figure 7(a)). We also searched the Human Protein Atlas database (https://www.proteinatlas.org/) and found that both SH3BGRL and ATG5 are relatively more highly expressed in normal tissues than their cancerous counterparts (Figure 7(b) and Figure S1C). Statistical analysis indicated that SH3BGRL is correlated to ATG5 expression level (Figure S1D), but not ATG12 level (Figure S1E) in liver cancers. To directly confirm the effects of SH3BGRL and autophagy occurrence in liver tumor progression, we performed immunohistochemistry and observed the positive relevance of SH3BGRL and autophagy event in xenograft mice tumors, in which the SH3BGRL expression is positively associated with autophagy proteins LC3 and ATG5 (Figure 7(c)). Taken together, our results solidly uncovered this novel SH3BGRL-ATG5-autophagy signaling axis in suppression of the liver tumor progression (Figure 7(d)).

## 4. Discussion

Recent evidence indicates that SH3BGRL acts as either a tumor metastasis suppressor [10, 11] or a tumor promoter [15], but the exact role and molecular mechanism of SH3BGRL in liver tumor progression remain unknown. Here, we unveil the promoting role of SH3BGRL in basal autophagy occurrence and its suppressive function in liver tumor progression and indicate that SH3BGRL would be a potential prognosis biomarker of liver cancer.

Mechanistically, SH3BGRL binds with ATG5 and stabilizes it to drive the basal liver cell autophagy through inhibition of Src and its downstream ERK and AKT signaling pathways that generally lead to tumor cell survival and proliferation. Thus, this novel SH3BGRL-ATG5 autophagy exerts a tumor suppression effect in liver cancer progression (Figure 7(e)). Meanwhile, this finding is consistent with the downregulation of SH3BGRL in liver cancers in our results and the public datasets. Therefore, inhibition of the downstream-activated signaling pathways would be an alternative therapeutic strategy for SH3BGRL-downregulated liver cancers.

Autophagy is an important physiological and biological process of cells through the intracellular self-degradation to maintain cell homeostasis and is recognized to often play dual roles in tumorigenesis and metastasis [19]. Usually, autophagy is induced by many environmental stresses such as hypoxia and nutrient deprivation. However, here we found that SH3BGRL can clearly trigger the basal liver cancer cell autophagy in a nutrient-sufficient situation, indicating the existence of basal level autophagy and its physiological function in cell homeostasis maintenance. Recently, we found that another nucleic acid-binding protein, PCBP1 exhibits a role in the suppression of basal level autophagy in ovary tumor cells under normal culture situations [20], collectively confirming the existence of basal autophagy of cells and its instinct balance regulation by functionally opposite factors in cells to sustain the cell homeostasis and prevent cells from over self-eating or overgrowth for the appropriate organism function. But both SH3BGRL and PCBP1 function as tumor suppressors with their overexpression, which challenges which type of autophagy is triggered or inhibited for tumor suppression or tumor promotion, and what is the biomarker to judge the eventual function of the dual autophagy effect in tumorigenesis? Therefore, the exact autophagy level of a cell and the cell context-dependency of a particular gene related to the regulation of autophagy should be further dissected.

ATG5 is an important protein in early autophagy formation, and ATG5 deficiency in melanocytes is associated with oncogene-dependent senescence-promoting melanoma tumorigenesis [21]. Likewise, deficiency of ATG5 effectively leads to tumorigenesis of liver and lung cancer in a mouse model [21]. Thus, the regulation of ATG5 is crucial for autophagy initiation and its subsequent functions. Here, we first unveil that SH3BGRL binds with ATG5 and stabilize it to trigger autophagy initiation, indicating the crucial physiological function of SH3BGRL in cell homeostasis, while linking the autophagy and tumor progression together. But the detailed underlying mechanism of SH3BGRL interaction with ATG5 would be further investigated, including the interaction among SH3BGRL, ATG5, and its E3 ligase that could lead to the competitive interaction between SH3BGRL and an E3 ligase to ATG5 and the ATG5 proteasome degradation process. Nevertheless, here we uncovered that SH3BGRL-induced autophagy functions as a tumorigenic suppressor to inhibit cell proliferation and cell cycle, indicating that enhancement of SH3BGRL depletion tumor with autophagy agonists to induce high-level autophagy would be beneficial to therapy. Moreover, the SH3BGRL-induced autophagy causes cell cycle arrest in liver cells and eventually leads to the suppression of tumorigenesis. This phenomenon of SH3BGRL-mediated inhibition of cell proliferation and arrest of the cell cycle can be strengthened under autophagic starvation conditions, which provides an alternative therapy for relevant cancers.

As an adaptor protein, SH3BGRL only contains two conventional binding motifs for the SH3 domain and a proline-rich motif and would connect multiple signaling cascades. It is documented that SH3BGRL can bind to EGFR, ErbB2, and ATG5 [17], but the physiological outcome needs to be investigated. Our results here demonstrate the interaction between SH3BGRL and ATG5 to promote autophagy and p62 degradation. Indeed, upregulation of p62 is commonly observed in human tumors, which directly contributes to tumorigenesis [22]. On another side, LC3 is shown to be positively correlated with long survival and lower hepatic cell cancer recurrence in patients, suggestive of the protective role of autophagy in liver tumorigenesis and tumor growth [23, 24]. Thus, the autophagy consequence and the detailed mechanism triggered by SH3BGRL overexpression should be extensively characterized based on the cell type and cell context concepts, which may be of help to disclose the underlying mechanism of the bidirectional effect of autophagy in tumorigenesis and tumor progression.

## 5. Conclusions

In conclusion, our results here first unveil the repressive role of SH3BGRL in liver tumor progression by enhancement of the basal autophagy of liver tumor cells, and highlight that SH3BGRL would be a potential prognostic marker for liver cancers, while either targeting the FAK-Src signaling or enhancing autophagy occurrence would be an effective strategy for the SH3BGRL-depleted liver cancers.

## Data Availability

All data supporting this study are provided in the main manuscript.

## Abbreviations

Act D: Actinomycin D
ATG5: Autophagy-related protein 5
CASP3: Caspase 3
CHX: Cycloheximide
CQ: Chloroquine
FBS: Fetal bovine serum
GAPDH: Glyceraldehyde-3-phosphate dehydrogenase
GEO: Gene expression omnibus
GFP: Green fluorescent protein
GSEA: Gene set enrichment analysis
IHC: Immunochemistry
KEGG: Kyoto Encyclopedia of Genes and Genomes
MAP1LC3B/LC3B: Microtubule-associated protein 1 light chain 3 beta
3-MA: 3-Methyladenine
SH3BGRL: SH3 domain binding glutamate-rich protein-like
SQSTM1/p62: Sequestosome 1
ULK1: Unc-51-like autophagy activating kinase 1.

## Additional Points

*Simple Summary.* Liver cancer is the most lethal malignancy without much efficacious therapy. Understanding the progression of liver cancer is the basis for effective therapy. Here, we unveil a small adaptor protein SH3BGRL that inhibits hepatic cell proliferation and tumorigenesis in a xenograft model. Mechanistically, SH3BGRL upregulation can stabilize ATG5 from degradation to promote autophagy-mediated liver cell death, while inhibiting Src activation and its downstream ERK and AKT signaling pathways. The relevance of SH3BGRL downregulation and liver cancer progression is also validated in patient samples. Thus, we first uncover a suppressive role of SH3BGRL in liver cancer progression, which would be useful for liver cancer diagnosis and the SH3BGRL-depleted liver cancers by either targeting the activated downstream signaling or enhancing autophagic liver cancer cell death.

## Supplementary Materials

Supplementary Materials Supplemental Figure S1: relevance of SH3BGRL and ATG5 in liver cancers. (A) Semiquantitative RT-PCR detection of SH3BGRL in the indicated liver cancer cells. *β*-actin was used as an internal control. (B) Semiquantitative RT-PCR detection of SH3BGRL mRNA expression in fresh liver tumors (T) compared to the normal tissues (N). *n* = 4, ^*∗*^*p* < 0.0298. (C) Immunohistochemistry of SH3BGRL and ATG5 from liver cancer tissues and the adjacent normal tissues from The Protein Atlas database (https://www.proteinatlas.org/). The sample ID and information are listed under each section. (D-E) The corelationship between SH3BGRL and either ATG5 (D) or ATG12 (E) is based on their protein expression level. Data are obtained from the public Cancer Genome Atlas (TCGA) dataset of liver cancers (https://www.cbioportal.org/results/coexpression?genetic_profile_ids_PROFILE_MUTATION_EXTENDED=lihc_tcga_pan_can_atlas_2018_mutations&genetic_profile_ids_PROFILE_COPY_NUMBER_ALTERATION=lihc_tcga_pan_can_atlas_2018_gistic&cancer_study_list=lihc_tcga_pan_can_atlas_2018&Z_SCORE_THRESHOLD=2.0&RPPA_SCORE_THRESHOLD=2.0&data_priority=0&profileFilter=0&case_set_id=lihc_tcga_pan_can_atlas_2018_cnaseq&gene_list=SH3BGRL%253B%2520ATG5&geneset_list=%20&tab_index=tab_visualize&Action=Submit).
