# Supplementary figures and images for: SH3BGRL Suppresses Liver Tumor Progression through Enhanced ATG5-Dependent Autophagy
Source: J Oncol. 2023 Apr 24;2023:1105042. doi: 10.1155/2023/1105042 (PMC10151150; doi:10.1155/2023/1105042)

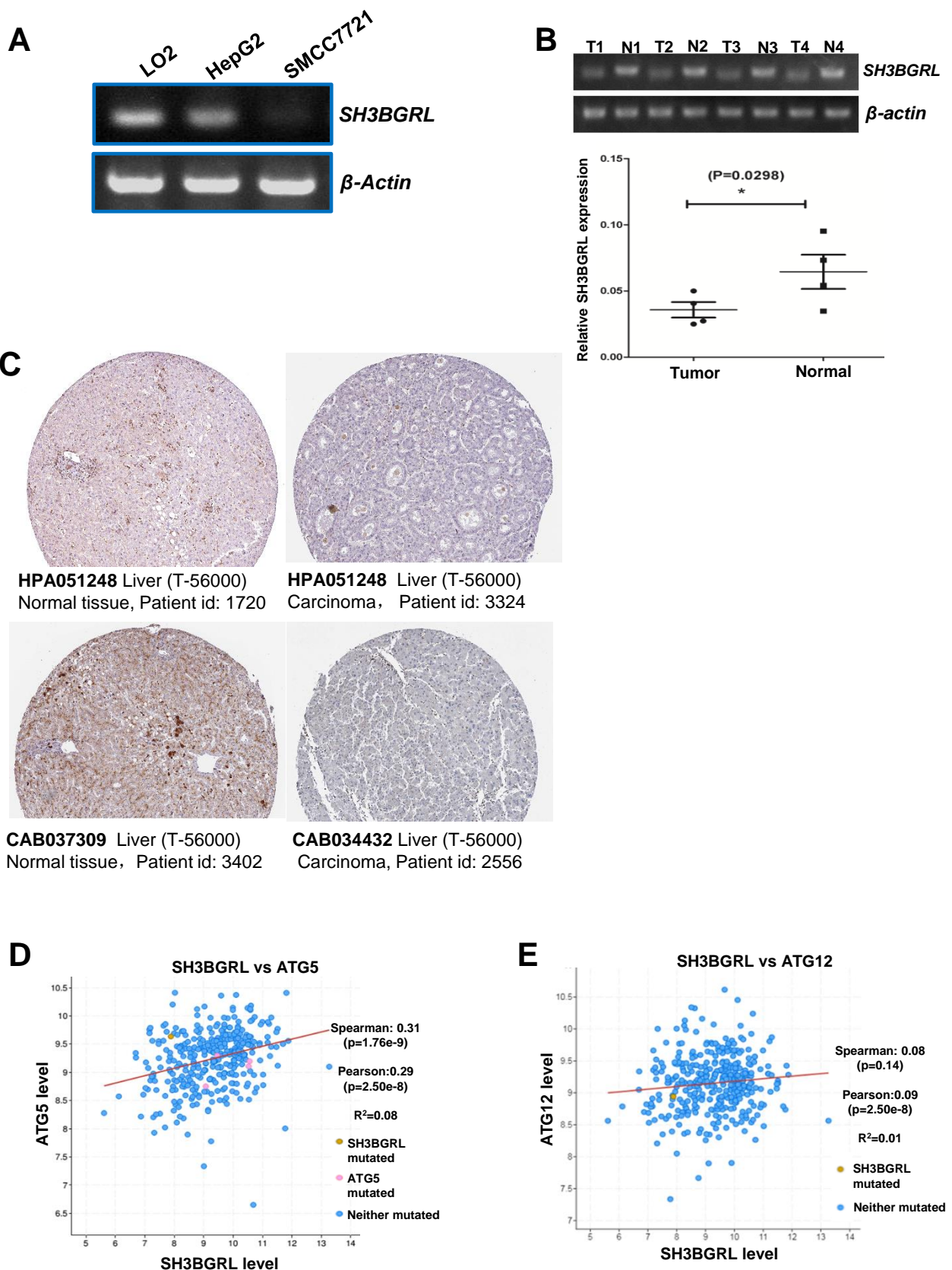

Supplemental Figure 1

Supplement: Supplementary Materials — Supplemental Figure S1: relevance of SH3BGRL and ATG5 in liver cancers. (A) Semiquantitative RT-PCR detection of SH3BGRL in the indicated liver cancer cells. β-actin was used as an internal control. (B) Semiquantitative RT-PCR detection of SH3BGRL mRNA expression in fresh liver tumors (T) compared to the normal tissues (N). n = 4, ∗p < 0.0298. (C) Immunohistochemistry of SH3BGRL and ATG5 from liver cancer tissues and the adjacent normal tissues from The Protein Atlas database (https://www.proteinatlas.org/). The sample ID and information are listed under each section. (D-E) The corelationship between SH3BGRL and either ATG5 (D) or ATG12 (E) is based on their protein expression level. Data are obtained from the public Cancer Genome Atlas (TCGA) dataset of liver cancers (https://www.cbioportal.org/results/coexpression?genetic_profile_ids_PROFILE_MUTATION_EXTENDED=lihc_tcga_pan_can_atlas_2018_mutations&genetic_profile_ids_PROFILE_COPY_NUMBER_ALTERATION=lihc_tcga_pan_can_atlas_2018_gistic&cancer_study_list=lihc_tcga_pan_can_atlas_2018&Z_SCORE_THRESHOLD=2.0&RPPA_SCORE_THRESHOLD=2.0&data_priority=0&profileFilter=0&case_set_id=lihc_tcga_pan_can_atlas_2018_cnaseq&gene_list=SH3BGRL%253B%2520ATG5&geneset_list=%20&tab_index=tab_visualize&Action=Submit). [file 1105042.f1.pdf]
